# Recent Advances in Micro/Nanomaterial-Based Aptamer Selection Strategies

**DOI:** 10.3390/molecules26175187

**Published:** 2021-08-26

**Authors:** Dong-Min Kim, Myeong-June Go, Jingyu Lee, Dokyun Na, Seung-Min Yoo

**Affiliations:** 1 Center for Applied Life Science, Hanbat National University, Daejeon 34158, Korea; dmk.iqbio@gmail.com; 2 School of Integrative Engineering, Chung-Ang University, Seoul 06974, Korea; mjaudwns@gmail.com (M.-J.G.); jingyu6611@gmail.com (J.L.)

**Keywords:** aptamer, SELEX, biomolecule, screening, affinity, specificity, library

## Abstract

Aptamers are artificial nucleic acid ligands that have been employed in various fundamental studies and applications, such as biological analyses, disease diagnostics, targeted therapeutics, and environmental pollutant detection. This review focuses on the recent advances in aptamer discovery strategies that have been used to detect various chemicals and biomolecules. Recent examples of the strategies discussed here are based on the classification of these micro/nanomaterial-mediated systematic evolution of ligands by exponential enrichment (SELEX) platforms into three categories: bead-mediated, carbon-based nanomaterial-mediated, and other nanoparticle-mediated strategies. In addition to describing the advantages and limitations of the aforementioned strategies, this review discusses potential strategies to develop high-performance aptamers.

## 1. Introduction

Specific recognition materials are vital in various fundamental studies and applications, such as biological analysis, disease diagnostics, targeted therapeutics, and environmental pollutant detection [1,2]. In clinical testing, these materials can detect specific disease markers and deliver a drug/biomolecule into the targeted cells. In an environmental setting, the ability of these materials to bind with specific pollutants can provide information related to the cause and degree of contamination. Their widespread use has prompted numerous efforts to develop materials with high affinities and specificities. Molecules such as antibodies, peptides, carbohydrates, enzymes, and chemicals have been developed, and among others, aptamers are artificial nucleic acid ligands with high affinity, specificity, high thermostability, acid-base resistance, low immunogenicity and toxicity, good tissue penetration, synthetic convenience, low cost, modifiable bioavailability, and small size (usually 6–30 kDa, ~2 nm in diameter) [3,4,5]. The aptamer can specifically bind various analytes, ranging from small molecules to macromolecules, cells, or tissues [6,7,8,9,10,11]. The scope of aptamer applications has recently been expanded to the fields of tissue staining, bio-imaging, and pharmaceuticals [12,13,14]. Diverse chemical and biomolecule-specific aptamers can also be commercially synthesized by companies (Table 1).

Aptamers can be discovered through an iterative in vitro process called systematic evolution of ligands by exponential enrichment (SELEX). Since the development of SELEX in 1990 [3,4], various types of SELEX have been further developed, including graphene oxide (GOx)-SELEX [15,16,17], capillary electrophoresis (CE)-SELEX [18,19,20], cell-SELEX [21,22,23], fluorescence-activated cell sorting (FACS)-SELEX [24,25,26], and photo-SELEX [27].

Several reviews have previously detailed the principles and applications of aptamer technology [28,29,30,31]. In this article, we focus on the recent advances in aptamer discovery strategies that have been used to detect various chemicals and biomolecules. These strategies rely on distinct characteristics of micro/nanomaterials such as magnetic beads (MBs), agarose beads, GOx, single-walled carbon nanotubes (SWNTs), and gold nanoparticles (AuNPs). These strategies have been discussed based on the classification of these micro/nanomaterial-mediated SELEX platforms into three categories: bead-mediated, carbon-based nanomaterial-mediated, and other nanoparticle-mediated strategies. Discussing the benefits and drawbacks of these techniques is useful for researchers, in both academia and industry, interested in the development and application of aptamers. Finally, future challenges and opportunities for aptamer discovery strategies are discussed and may enable the development of high-performance aptamers.

## 2. Aptamer Discovery Strategies for Molecule Detection and Monitoring

### Basic Process of Aptamer Discovery

Aptamers are short, single-stranded DNA (ssDNA), RNA (ssRNA), or XNA (xeno nucleic acid, which is a synthetic nucleic acid analogue) [32,33] molecules that can selectively bind to a specific target. The presence of a target induces a conformational change in the oligonucleotide, facilitating an interaction between the aptamer and the target. Aptamers with an affinity for a desired target can be selected through SELEX. SELEX begins by screening a large oligonucleotide pool to identify an aptamer with a high affinity against the target and a low cross-reactivity against the counter target. This oligonucleotide usually consists of two parts: a randomly generated sequence and a constant sequence. The random sequence is the region that binds with the target molecules and is located in the middle of aptamer flanked by the conserved sequences that are the regions that bind with the polymerase chain reaction (PCR) primers. The conserved regions with a constant sequence are required for the primer-driven PCR amplification of the oligonucleotide pools for the next round of SELEX. 

With these constituents, the oligonucleotide pools can be designed to exhibit an unstructured, linear form. Additional constant sequences can be placed in the middle of the random sequence (generally 30–50 randomized nucleotides) to bind functional materials such as MBs, enabling the simple and easy separation of target-bound or counter target-bound pools. Alternatively, pools can be designed to have diverse three-dimensional structures within a fixed length. The secondary structures of aptamers provide several advantages compared to an unstructured aptamer, such as thermodynamic and chemical stability, low immunogenicity, and resistance to nucleases [34]. In order to form the structure, these parts must contain additional sequences. For example, aptamers with a hairpin structure have two portions that are complementary to each other and can form base pairing. The kissing complex-forming aptamer, which has been proposed to improve affinity and specificity, has a few unpaired bases of one hairpin loop and its complementary unpaired bases in another hairpin loop, leading to multiple loop–loop interactions between adjacent hairpins [35,36,37]. The G-quadruplex-forming aptamer has stacked G-tetrads connected by loop residues that are stabilized by target molecules within the central cavity, exhibiting a highly polymorphic structure [38,39,40,41,42]. 

Pre-enriched aptamer pools are then exposed to target molecules. Unbound aptamers are discarded, and target-bound aptamers are collected. The separation of target-bound aptamers is a critical step to identify specific and sensitive aptamers. There have been many efforts to develop diverse partitioning strategies. One strategy is to design aptamers for the desired purpose, which can determine whether unbound or target-bound aptamers are captured. For example, aptamer pools with a defined docking sequence in the random region can form a duplex by binding to oligos with sequences complementary to the docking region of the aptamers on materials, resulting in being indirectly immobilized on materials [43]. When an aptamer binds to a target molecule, its conformational change causes it to be released from the materials, enabling the collection of target-bound aptamers. This method of using aptamer immobilization is more suitable for small molecule targets than the target immobilization method. Another strategy is to employ specific properties of certain materials. For instance, random ssDNA aptamer pools have been mixed with target molecules and then incubated with AuNPs [44]. AuNPs absorb only free ssDNA [45]; therefore, the AuNPs-absorbed target-unbound aptamers can be separated by centrifugation from the target-bound aptamers. The surface of GOx also strongly absorbs free ssDNA through hydrophobic and π–π stacking interactions between the nucleobases of the ssDNA and the GOx [46,47,48,49]; therefore, upon the exposure to target molecules, the unbound aptamers bind to GOx, while the target-bound aptamers remain in the solution. After centrifugation, ssDNA-bound GOx can be removed, and target-bound aptamers can be recovered [50,51]. 

The collected target-bound aptamers are amplified by PCR for the next round of SELEX. Aptamers can be often eluted by releasing free aptamers from target-bound aptamers through exposure to the target in a high concentration, high temperature, and a detergent [52,53,54].

The removal of non-binders and the screening of binders are iteratively accomplished by monitoring the aptamer-target binding in every round until the aptamers with the desired sensing performance are selected. Before the next round, the amplified dsDNAs should be prepared in the form of ssDNA or ssRNA aptamers. In order to construct this ssDNA form, dsDNAs can undergo various processes such as asymmetric PCR, enzyme-assisted cleavage reaction, streptavidin–biotin chemistry, denaturation gel electrophoresis, and rolling circle amplification [55,56,57]. For ssRNA, reverse transcription PCR is applied. After cycling selection rounds, the enriched pools are sequenced to define the aptamers and further evaluate their characteristics, such as structure, binding affinity, and binding site of the target. 

## 3. Current Aptamer Discovery Strategies to Detect Chemicals and Biomolecules

Table 2 summarizes examples of micro/nanomaterial-based SELEX platforms, including those described in this review and others, that have been employed for the selection of aptamers for various molecules. Table 3 summarizes the advantages and limitations of the strategies. 

### 3.1. Bead-Mediated Aptamer Selection

There are many beads made of various materials, such as magnet, nickel, and agarose. Utilizing the distinct characteristics of these beads can enhance the performance of SELEX.

MBs are among the representative beads used in SELEX that enable simple and rapid partitioning of target-bound and unbound aptamers through the use of a magnet [51,58,62,81,82]. The quenching of MBs against fluorescence [83,84] can also be used for aptamers. The immobilization by diverse chemical reactions requires an additional functionalization process for either the bead or the oligonucleotide. The strategy using MBs to separate target-specific aptamers is further divided into two sub-strategies: target-immobilization on MBs and aptamer-immobilization on MBs. Both strategies require extensive and tedious conjugation processes and suffer from steric hindrance between targets and aptamers, potential nonspecific adsorption, and possible conformational changes [50]. For the strategy involving immobilization of target molecules on MBs, the target-immobilized MBs can directly capture the aptamers with high affinity toward the target without non-specific adsorption through a stringent washing step. However, as the target is pre-immobilized on the MB using its inherent functional group, the MB can cause difficulty or even failure of the selection by blocking an important binding site and/or through steric hindrance in the binding of the aptamer and the target, especially for small molecules. Therefore, a potential structural alteration of the target after immobilization on beads, which may lower the screening efficiency of aptamers, should be considered.

**Figure 1:**
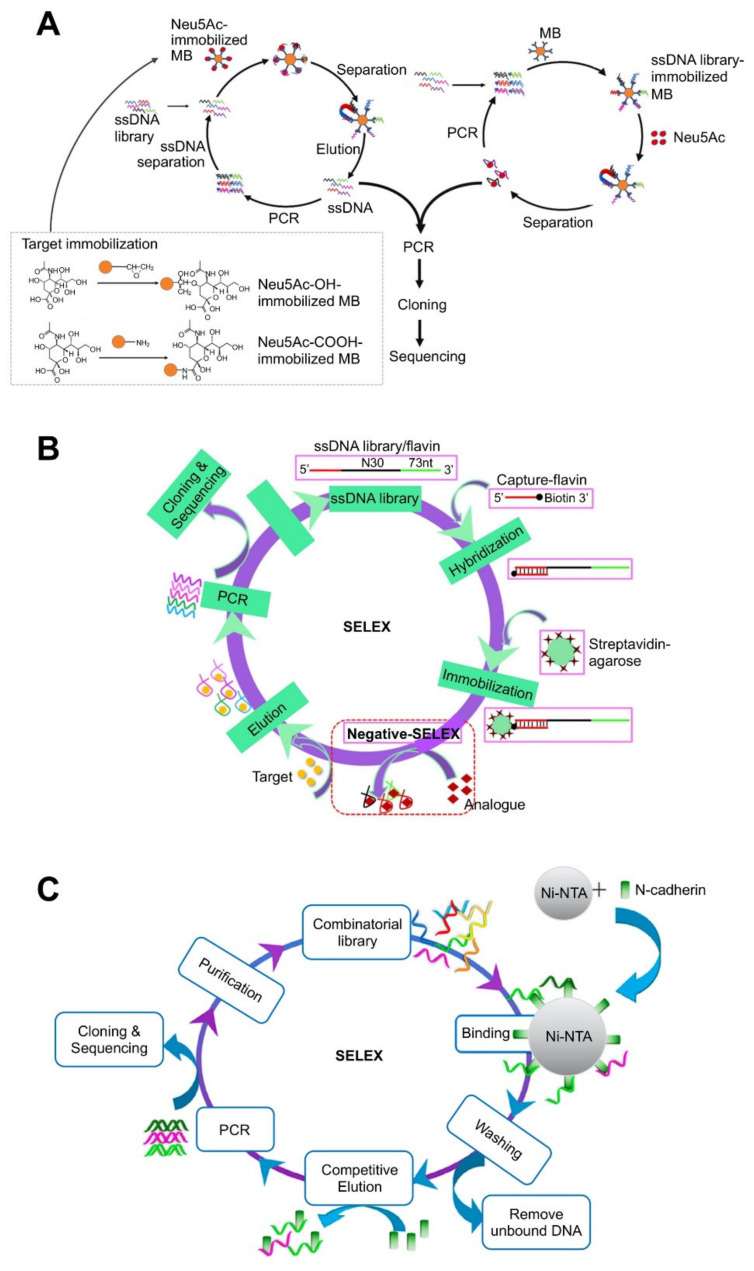
Aptamer discovery strategy using bead–based platforms: (**A**) N-acetylneuraminic acid (Neu5Ac)-specific aptamer selection using a magnetic bead platform. Reproduced with permission from [58]; (**B**) Riboflavin-specific aptamer selection using an agarose bead platform. Reproduced with permission from [64]; (**C**) His-tagged N-cadherin protein-specific aptamer selection using a nickel Sepharose bead platform. Reproduced with permission from [70].

Immobilizing oligo pools onto MBs is an alternative strategy. For example, biotin-labeled aptamers directly bind to streptavidin-coated MBs [63] or biotin-labeled capture probes first bind to streptavidin-coated MBs and the aptamer pools then bind to the capture probes on the MBs by base-pairing [85]. This strategy allows a relatively free interaction between the aptamer and the target, which makes it particularly suitable to screen for small molecules. However, in this strategy, oligos need to be either modified to tether onto the matrix or designed to have sequences complementary to a docking region on the target. Steric hindrance can also result in a collection of non-specific aptamers along with limited binding; therefore, an additional screening step is required; negative selection using a counter target can overcome this limitation. The pre-enrichment of aptamers by additional PCR amplification is usually required before aptamer immobilization on the MBs. A recent study has compared these two strategies by screening aptamers for a low-molecular target: N-acetylneuraminic acid (Neu5Ac) (Figure 1A) [58]. In this target-immobilized strategy, the MBs were first functionalized with epoxy and amino groups, which can bind to the hydroxyl and carboxyl group of Neu5Ac, respectively. After the addition of targets, target-bound aptamers were separated by a magnet in each round; the finally selected aptamers were validated by sequencing after the last round. For the aptamer-immobilized MB strategy, aptamer pools were first amplified with biotin-labeled primers and the resultant dsDNAs were immobilized onto streptavidin-coated MBs. The strands with a high affinity for the target detached from their complementary strands on the MBs; the strands released in the solution were isolated by removing the MBs with a magnet. The selection efficiency and average affinity of aptamers for Neu5Ac were higher in the aptamer-immobilized strategy. The best aptamer for Neu5Ac had a *K*_d_ value of 55.71 ± 12.29 nM. The target or library immobilization strategy can be also applied when using other micro/nanomaterial-based platforms.

Agarose beads have also been used as SELEX platforms and used to select aptamers for diverse chemicals [64,65,66,69,86]. Agarose beads are linear hydrogel polymers comprising repeated units of agarobiose. They have been widely used in packed columns for size exclusion or affinity chromatography. In the SELEX process, agarose beads with functional groups are packed in a column to anchor the targets or the aptamer pools on the matrices. For example, to screen aptamers specific to riboflavin and flavin adenine dinucleotide (FAD), unmodified aptamer pools were first hybridized with biotinylated capture probes, followed by immobilization on streptavidin-coated agarose resins packed in a column (Figure 1B) [64]. After incubation with the targets, the ssDNAs with a high affinity can bind to the target, inducing conformational change and releasing from the resins. In order to achieve high selectivity, SELEX was performed first for negative selection and then for positive selection. The finally selected aptamers exhibited a *K*_d_ value of 0.61 ± 0.04 against riboflavin and 0.44 ± 0.02 against FAD. 

Another study has used agarose beads to screen aptamers with high affinities for three targets at once: ethylone, butylone, and alpha-pyrrolidinopentiophenone (α-PVP) [70]. ssDNA pools were first bound to biotinylated capture probe DNAs, followed by a transfer to columns packed with streptavidin-coated agarose beads. Since the three target molecules have the same core structure, SELEX for each target was proceeded parallelly, after which the three aptamer pools were combined. A serial selection process was applied to the combined aptamer pools involving the sequential addition of three targets and the elimination of only one target-bound ssDNA in each process. The selected aptamers exhibited affinities with *K*_d_ values of 6.9, 9.5, and 21 μM for ethylone, butylone, and α-PVP, respectively. 

The aptamer for Lucentis was selected by immobilizing the target on the agarose beads [66]. An aptamer with high affinity was obtained by decreasing the concentration of the immobilized target and the incubation time, increasing the number of washing steps, as well as expanding the elution procedure, which could reduce the number of cycles. Within 10 SELEX cycles, an aptamer with a *K*_d_ value of 25.72 ± 4.2 nM was obtained. The aptamers for (−)-trans-Δ9-tetrahydrocannabinol (THC), XLR-11, and UR-144 were screened using oligo library-immobilized agarose beads [67] to freely bind to their natural targets without any deformation during target immobilization, which is necessary for small molecules with only a few functional groups for aptamer binding. These three targets are highly hydrophobic; therefore, a binding buffer containing an organic solvent (i.e., 2.6% methanol or 5% DMSO) was used during the SELEX process to increase their solubility. With unmodified ssDNA, the aptamers with high affinities and *K*_d_ values of 61 ± 25 nM (toward THC), 310 ± 70 nM (toward XLR-11), and 127 ± 32 nM (toward UR-144) were selected. 

An aptamer for 3,4-methylenedioxypyrovalerone (MDPV), a small molecule, was also selected using library-immobilized agarose beads [68]. This selection utilized a three-way junction (TWJ)-forming oligo library that could be stacked with diethylthiatricarbocyanine (Cy7). Using a TWJ-structured library results in a more accurate selective assay involving dye displacement instead of the strand-displacement strategy. In the strand-displacement assay, when the complementary strand-selected aptamer duplex meets the target, the preferred binding of the aptamer to the target leads to a release of the aptamers from the duplex. However, for complementary strands with a high binding affinity for the aptamer, strand displacement does not occur. This weak reaction can occur frequently in small molecules, consequently reducing the selectivity of the aptamer. In the dye-displacement assay, when the target is added, the complementary strand-selected aptamer duplex maintains its structure and the target is incorporated into the duplex instead of Cy7. This assay can be used without further oligo engineering.

Nickel beads have also been used to screen aptamers for the N-cadherin protein (Figure 1C) [70]. This system relies on an interaction of histidine and Ni (II), which has been widely exploited for protein purification by his-tagging target proteins. N-cadherin was first modified with polyhistidine at the C-terminus and subsequently immobilized onto Ni Sepharose beads in a packed column. Upon the addition of aptamer pools, unbound DNA strands were removed by washing and the target-bound DNA strands were detached from the bead by adding the N-cadherin, resulting in the column-captured DNA strands competing with the added external targets, and eventually being eluted out from the column. Using this system, an aptamer with a *K*_d_ value of 93 nM was selected. This tag can act as a linker to lift targets on a solid substrate; therefore, tag-mediated immobilization might allow more free binding to aptamers than that by the functional group of the protein itself, wherein some domains of the target can be blocked. The required additional his-tagging of the target can limit the spectrum of diverse targets that can be identified in this system. 

### 3.2. Carbon Nanomaterial-Mediated Aptamer Selection

GOx is single atomic layer of carbon with various oxygen-containing functionalities. SWNT is a graphene layer seamlessly rolled into a tube. These two materials can adsorb ssDNA or ssRNA through π−π stacking interactions between the hexagonal cells of GOx or SWNT and the ring-like structures in the DNA bases [46,47,48,49]. GOx and SWNT are generally used for the easy and full partitioning of unbound or target-bound oligonucleotides, which enhances screening efficiency [71,73,87]. The adsorption of single-stranded oligos enables the removal of unbound oligos without any immobilization of the target or the aptamer on the substrate, thereby overcoming some major limitations (i.e., steric hindrance, additional functionalization of oligos, etc.) of immobilization SELEX mentioned in Section 3.1. Non-immobilization strategy allows for binding between oligos and targets in the natural state without any complicated surface interference.

**Figure 2:**
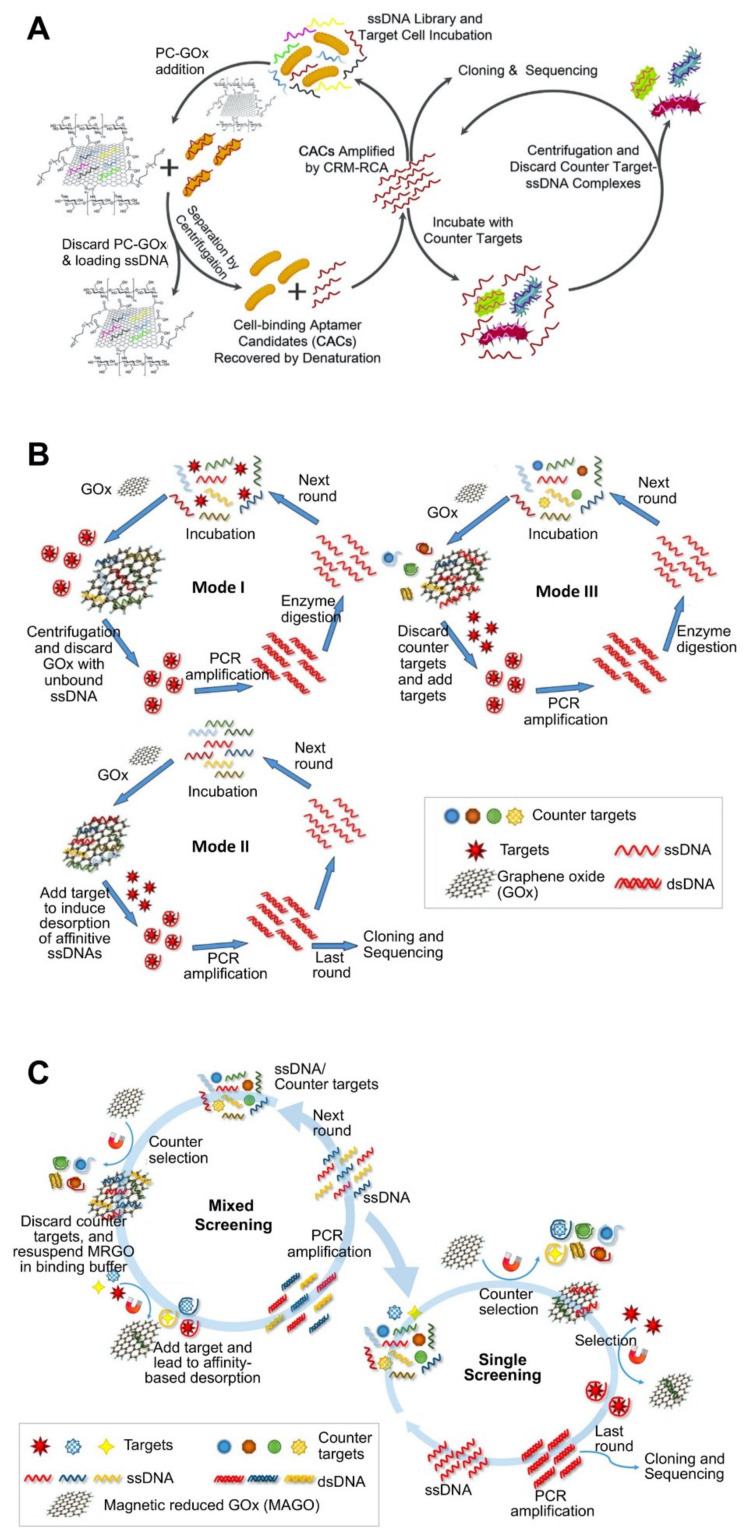
Aptamer discovery strategy using carbon nanomaterial-based platforms: (**A**) *Vibrio parahaemolyticus*-specific aptamer selection using a polyethyleneglycol and chitosan (PC)-functionalized graphene oxide (GOx) platform. Reproduced with permission from [71]; (**B**) Okdaic acid-specific aptamer selection using a GOx platform. Reproduced with permission from [72]; (**C**) Saxitoxin, domoic Acid, and tetrodotoxin-specific aptamers selection using a magnetic reduced GOx (MAGO) platform. Reproduced with permission from [51].

Based on its distinct characteristic of adsorbing single-stranded oligos, the GOx-based SELEX process can be performed by the three strategies. In the first strategy, oligo pools and the target are mixed, and the added GOx captures target-unbound oligos and discards them by centrifugation. The target-bound oligos in the solution are amplified and prepared in the form of single-stranded oligos for the next round. However, this strategy may have limited applications for macromolecules such as cells because the limited water solubility of GOx and SWNT can cause co-precipitation of GOx-adsorbed unbound oligo with target cells, leading to failed separation. In order to overcome this limitation, a modified SELEX strategy using GOx with new functionality was developed, wherein GOx was functionalized with polyethyleneglycol (PEG) and chitosan (CTS) (Figure 2A) [71]. The introduction of PEG increases the solubility of GOx, whereas that of CTS makes the GOx-based platform more biocompatible with cells. In the second strategy, oligo pools and the counter target are mixed; the added GOx captures target-unbound oligos, isolating them by centrifugation. After adding the target, the oligos bind to it and undergo conformational changes, thereby being released from GOx and then proceeding to the next round.

The third strategy is to use GOx as an oligo-immobilizing platform like in the bead-based strategy. The added target induces the release of target-bound oligos from the oligo pools immobilized on GOx. Through centrifugation, target-bound oligos in the solution were selected for the next round. Unlike other materials, GOx can immobilize these oligos without any modification or use of functional groups. By applying these three strategies, okadaic acid (OA)-specific aptamers were screened (Figure 2B) [72]. In this study, OA is too small to bind with a low number of affinitive oligos; therefore, the SELEX process was performed by applying three strategies sequentially: the oligo-free strategy was applied for the early rounds, the oligo-immobilizing strategy was employed for the later rounds, and a counter-selection strategy was used for the final few rounds. This study suggests that small molecules may not have a chance to bind to the target; therefore, the order of these strategies is important to screen the oligos satisfactorily.

Recently, another non-immobilized magnetic-reduced GOx (MRGO)-assisted SELEX has been developed and applied to screen aptamers for the following three small molecules: domoic acid (DA), saxitoxin (STX), and tetrodotoxin (TTX) (Figure 2C) [51]. The interaction of aptamers with the target molecules occurs in the solution. This system uses MRGO with the properties of MBs and GOx as the aptamer screening platform to facilitate simple partitioning and enhanced binding between the aptamer and the target molecule. The target-bound aptamer remains in the solution, while the unbound aptamer can be captured by MRGO and removed using a magnet. Similar to the GOx-based aptamer immobilization method, this system starts by mixing aptamer pools with the target molecules without any immobilization. Unlike the GOx-based aptamer immobilization method that uses centrifugation-based separation, this system uses MRGO to simply separate aptamers using a magnet. For a single selection, a counter selection was first performed and the target molecules were subsequently added, whereas for the selection of multiple aptamers, multiple target molecules were added, and then counter molecules were exposed. Using this system, the finally selected aptamers for DA, TTX, and STX exhibited low *K*_d_ values of 62.07 ± 19.97, 44.12 ± 15.38, and 61.44 ± 23.18 nM, respectively. 

In addition to single-stranded oligo-adsorption, fluorescence quenching is another distinct property of GOx [16,71]. GOx acts as a quencher for most florescent materials in a broad range of absorption spectra [88,89,90]. Its high fluorescence quenching efficiency and low background signal (i.e., low noise) have also been utilized to develop fluorescence resonance energy transfer sensors [88]. Aptamers for sulfaquinoxaline (SOX) were selected using TAMRA-labeled oligos and GOx [16]. TAMRA-labeled oligos were mixed with SOX, and the added GOx was then bound by the free oligos and removed by centrifugation. Optimal SELEX conditions, such as GOx concentration and incubation time, were determined by monitoring the decrease or increase of the signal caused by fluorescence quenching of GOx. The affinities and specificities of the selected aptamers were also validated in the same manner. Upon exposure to the target, aptamers with high affinity and specificity bind to the target and undergo conformational changes, which prevents them from binding to GOx, thereby maintaining the fluorescence signals emitted by aptamers. When the counter target is added or the aptamer has low affinity and specificity, the interaction of the target and aptamer does not occur, and the free aptamer is captured by GOx, thereby leading to a decrease in the signal intensity of the aptamer.

### 3.3. Other Nanoparticle-Mediated Aptamer Selection

**Figure 3:**
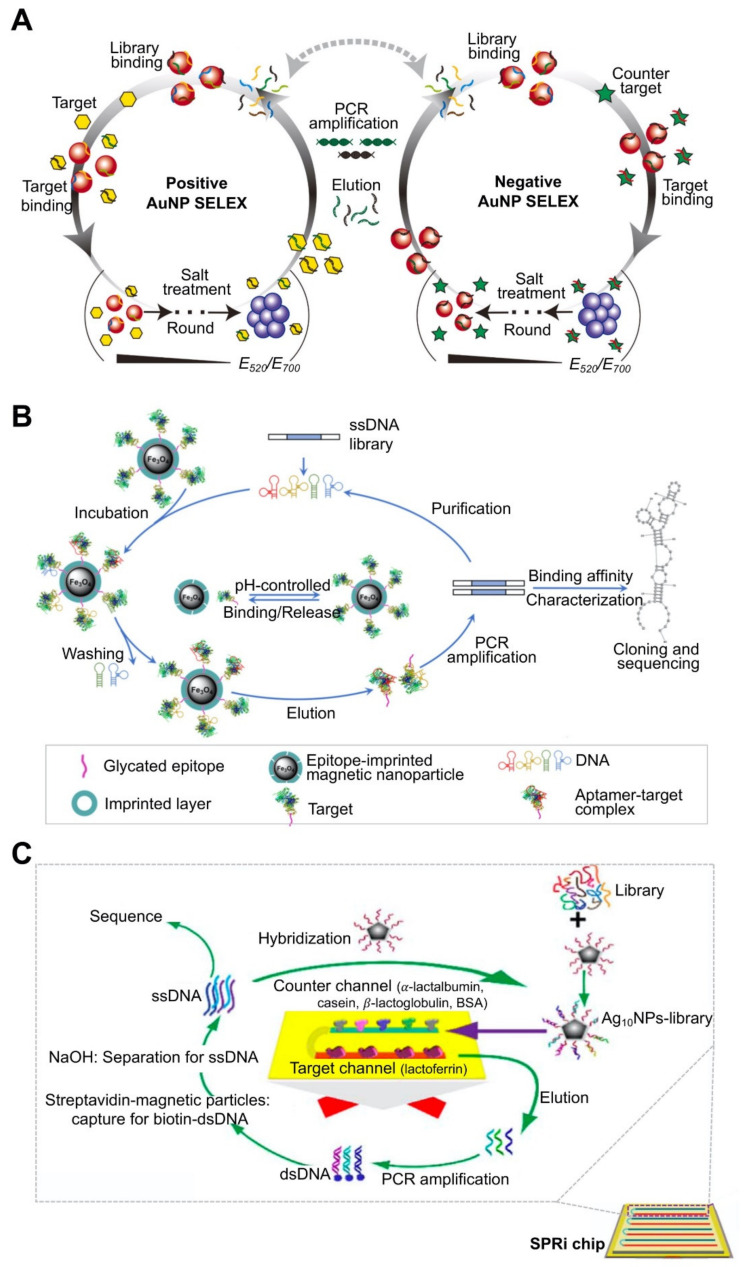
Aptamer discovery strategy using other nanomaterial–based platforms: (**A**) Brassinolide and bisphenol A-specific aptamers selection using a gold nanoparticle (AuNP) platform. Reproduced with permission from [74]. (**B**) Myoglobin and β2-microglobulin-specific aptamers selection using an epitope-imprinted magnetic nanoparticle platform. Reproduced with permission from [78]; (**C**) Lactoferrin-specific aptamer selection using a silver decahedral nanoparticle (Ag_10_NPs)-based surface plasmon resonance imaging (SPRi) platform. Reproduced with permission from [80].

Nanomaterials have unique electronic, optical, and magnetic characteristics [91]. Among the various nanomaterials, AuNPs have the same properties of DNA adsorption and fluorescence quenching that are possessed by GOx [44,75,92,93]. For instance, AuNPs can capture free oligos and selectively remove target-unbound oligos from a target-mixed ssDNA library [44]. Using AuNPs has several advantages compared to using GOx. One advantage is that it enables efficient monitoring of the SELEX progress without requiring additional analytical methods, such as real-time quantitative PCR or next-generation sequencing [60,94,95]. This advantage is attributed to the distinct characteristic of AuNPs. Spherical AuNPs exhibit a range of colors (e.g., brown, orange, red, and purple) in aqueous solutions depending on their size. However, the addition of NaCl, a strong electrolyte, shields the negative cores of the colloidal AuNPs and causes them to clump together to form aggregates, resulting in color changes, whereas single-stranded oligo-bound AuNPs maintain their intrinsic colors [89]. Leveraging this property of AuNPs, a recent study reported an effective method to monitor SELEX progress using salt-induced NP aggregation (Figure 3A) [74]. In this system, ssDNA pools were first immobilized on AuNPs using the van der Waals forces between the hydrophobic nitrogen bases of the oligos and the AuNP surface. Upon addition of the target, aptamers with high affinity for the target bind to the target and then are detached from the NPs; subsequently, the free NPs aggregate in the presence of salt in the buffer, resulting in color changes under high-salt conditions. Aptamers with high affinity can be collected from the supernatant by centrifugation and used for the next round of SELEX. In order to obtain an aptamer with high specificity, a counter target is used in this system. The SELEX process to remove the non-specific oligos can be monitored in real-time by comparing the color change of the positive SELEX using the target molecule to that of the negative SELEX using the counter target.

Magnetic nanoparticles (MNPs) are another type of nanoparticle that has been used as a SELEX platform to identify aptamers against bacteria, glycoprotein, and other proteins [92,93,94,95]. The magnetic property of MNPs enables the simple and facile partitioning of oligos following the incubation of the target with the oligos. Recently, MNPs have been combined with a molecularly-imprinted polymer (MIP) to endow functionality to the SELEX platform [76,77,78,79]. MIPs are artificial molecules that specifically bind to template molecules. The fabrication process for MIPs is initiated by the polymerization of monomers upon exposure to template molecules. This molecular imprinting technique leaves a cavity in the polymer matrix, which is the binding region for the target molecules functioning as a lock and key mechanism, thereby ensuring the high selectivity of MIPs [96]. The introduction of MIPs enables an interaction between the MNPs and the target, while retaining the target’s native properties and structure because their binding occurs through this imprinted cavity and not a functional group of the target. This can overcome the limitation of conventional target-immobilizing strategies.

Using MIP-combined MNPs, target-immobilizing SELEX was developed to screen aptamers for two glycoproteins: RNase B and transferrin [77]. This system uses glycan-imprinted MNPs as the SELEX platform. This platform was prepared by functionalizing boric acid-coated MNPs with N-linked glycans, which were prepared by digesting the target glycoprotein using PNGase F. Boric acid on the MNPs can covalently bind with cis-diol-containing compounds such as glycoproteins in basic solutions, whereas this formed complex can dissociate in an acidic solution [97,98]. Therefore, this platform facilitates specific interaction of target glycoproteins depending on their pH level: at a high pH (>7.0), glycans bind to the glycoprotein, forming a complex, and at a low pH (<3.0), their binding is degraded, thereby dissociating the complex. When target-anchored glycan-imprinted MNPs are incubated with ssDNA pools, oligos with high affinities for the target glycoprotein bind to the MNPs. Pure oligos are obtained from MNPs by treating with them acidic solution (0.1 M HAc solution) for the next round of SELEX. This pH-induced binding/dissociation reaction ensures simple and efficient recovery of target-bound aptamers from MNPs. Binding abilities and affinities for the target could be improved further by functionalizing MNPs with polyethyleneimine, which forms a branched platform to bind more boronic acid moieties on the MNPs [79]. This platform could screen aptamers for two saponins (ginsenoside Re and Rb1) with *K*_d_ values of 2.3 ± 0.3 (for Re), 4.9 ± 0.3 μM (for Rb1).

In a similar strategy, in order to screen aptamers for myoglobin (Mb) and β2-microglobulin, epitope-imprinted MNPs have been developed (Figure 3B) [78]. Epitope imprints comprise C- or N-terminal peptides of the target proteins, thereby enabling specific binding with their epitopes. The facile process of this SELEX is attributed to the magnetic property of MNPs and the pH-controlled capture/release of oligos by the epitope-imprinted MNPs. Using this platform, aptamers for Mb and β2-microglobulin were screened within a short period of time (three cycles, 1 day), exhibiting *K*_d_ values of 46.3 ± 10.4 and 36.7 ± 11.4 nM, respectively. The short cycling can lower potential limits such as loss of rare sequences, PCR bias, and retention of non-specific and low affinity aptamers.

Silver nanoparticles (AgNPs) have also been used for SELEX (Figure 3C) [80]. AgNPs exhibit strong plasmon resonance and have therefore been used extensively to label molecules using various surface plasmon resonance (SPR)-based techniques [99,100]. Using this property of AgNPs, AgNP-based SPR imaging (SPRi)-SELEX has been developed and used to select an aptamer for lactoferrin (Lac) [80]. An ssDNA library was attached to AgNPs with decahedral structures. The target, Lac, and counter targets were immobilized on different channels of the Au film, which was equipped with multi-channel configuration, enabling the high-throughput detection of samples. The selected aptamer exhibited a *K*_d_ value of 0.953 ± 0.114 nM.

## 4. Conclusions and Future Perspectives

Over the past decade, ssDNA, ssRNA, and XNA molecules, termed aptamers, have typically been screened from a random sequence library in vitro by the SELEX process. Aptamers are being increasingly used as bio-recognition ligands for practical applications in industrial, environmental, and clinical settings. This widespread use is attributed to their high affinity, specificity, good stability, ease of synthesis and modification, low cost, and small size. This review focused on recently developed aptamer discovery strategies that employ micro/nanomaterials as the SELEX platform and describes recent applications of screening aptamers with high affinities and specificities toward various target molecules. Although tremendous progress has been made in developing SELEX strategies, several challenges remain to be overcome.

First, the recognition/binding sites of the target molecules for the aptamers need to be clearly identified for accurate detection results and wide application. For instance, the target binding spectra of these aptamers are often either insufficient or unpredictable in screening for small molecules because of their small sizes and similar core structures. Macromolecules such as cells and tissues are complex; when an aptamer is selected, its specificity not only depends on structural conformation, but also on the in vivo cellular environments. Many software packages have been developed to predict and simulate the structure of aptamers that are selected by the SELEX process or the interaction between the aptamer and target before SELEX is completed (Table 4). Most secondary structure prediction algorithms are based on calculating minimal free energy and/or portioning functions. As aptamers have secondary (2D) structures, such as a hairpin and G-quadruplex structure, to improve affinity and specificity, as mentioned above, the prediction of 2D structures is essential to understand interaction between aptamer and target molecules. When aptamers are attached to micro/nanoparticles to achieve diverse purposes, the precise 2D structure prediction is very important. The 2D structure information also plays a vital role in aptamer tertiary (3D) structure prediction; practically, most 3D structure prediction algorithms use 2D structure elements as input data. When an aptamer binds to target proteins to have diverse functions, aptamer 3D structure information is important to predict and evaluate the function and the mode of action of these complexes. Molecular docking can be a crucial tool to predict the predominant binding mode and binding sites of the proteins and the ligands. The representative docking tools used for aptamer design are ZDOCK, MDockPP, AutoDock, and AutoDock Vina. Computer simulation and prediction might be necessary to predict and analyze the binding sites of existing aptamers specific to target molecules.

Second, simple and rapid processes should be optimized. An iterative process may be essential to select aptamers with high affinity and specificity for target molecules. However, this repetitive process is time-consuming and cost-intensive; therefore, an optimal SELEX cycle is needed. Although increasing the number of cycles results in a saturated selection, excessive selection rounds can lead to the selection of false-positive oligos. This challenge was overcome by incorporating a microfluidic system for fast and automatic operation of this SELEX process, which includes incubation, expression, and amplification. This system has been demonstrated to successfully screen aptamers for ovarian cancer cells, ovarian cancer tissue, the monoclonal Fab region of immunoglobulins, and cholangiocarcinoma cells [11,118,119,120]. 

Third, the efficiency of selected aptamers should be validated in vivo. In the past several decades, many aptamers have been selected for various target molecules. However, many exhibit poor specificity and affinity, a short half-life, and susceptibility to nuclease present in the cell and blood, which limits their widespread applications in vivo. A recent study reported that nanocomposites and hybrid aptamer-immobilized GOx effectively inhibit thrombin’s coagulant activity in the blood [121]. This hybrid aptamer comprises two different aptamers (a 15-mer and a 29-mer) against thrombin that are linked with multiple poly (adenine) (A_20_) segments. As GOx preferentially binds to single-stranded oligos with adenosine [122], the A_20_ segment allows the aptamer to bind strongly to the GOx, thereby ensuring high stability of these composites in vivo. 

With such advances, we believe that micro/nanomaterial-based SELEX strategies will continue to evolve and facilitate highly specific and sensitive aptamer discovery, thereby increasing their practical application in industrial and biomedical settings.

## Figures and Tables

**Table 1:** Examples of aptamer development companies.

Company	Website
Optimer™	https://aptamergroup.com/ (accessed on 24 August 2021)
Creative Biolabs	https://www.creative-biolabs.com/ (accessed on 24 August 2021)
Oak Biosciences	https://www.oakbiosciences.com/ (accessed on 24 August 2021)
TriLink Biotechnologies	https://www.trilinkbiotech.com/ (accessed on 24 August 2021)
Novaptech	https://novaptech.com/ (accessed on 24 August 2021)
BasePair Biotechnologies	https://www.basepairbio.com/ (accessed on 24 August 2021)
Aptagen	https://www.aptagen.com/ (accessed on 24 August 2021)
Aptamer sciences	http://aptsci.com/en/about-us/overview/ (accessed on 24 August 2021)
Novaptech	https://novaptech.com/ (accessed on 24 August 2021)
AptaTargets	http://aptatargets.com/ (accessed on 24 August 2021)
RIBOMIC	https://www.ribomic.com/eng/technology.php/ (accessed on 24 August 2021)
NeoVentures Biotechnology	https://neoaptamers.com/ (accessed on 24 August 2021)
TAGCyx Biotechnologies	https://tagcux/com/en/ (accessed on 24 August 2021)
Raptamer	https://raptamer.com/ (accessed on 24 August 2021)

**Table 2:** Examples of micro/nanomaterial-based selection of aptamers for various target molecules.

Classification	Material	Target Molecule(s)	*K*_d_ Value	*K*_d_ Value-Measuring Method	Characterization Method	Features	Reference
Bead-based platforms	Magnetic bead (MB)	N-acetylneuraminic acid	55.71 ± 12.29 nM	Fluorescence assay by labeling aptamer with FAM at the 5′-end	Prediction of two-dimensional (2D) structure using Mfold. Three-dimensional (3D) structure analysis by RNAComposer.	Use of both immobilization strategies using target-immobilized MBs and aptamer-immobilized MBs.	[58]; Figure 1A
	MB	PCSK9	70 ± 10 nM	Biolayer interferometry assay	Target-binding analysis by qPCR and chemiluminescence.	Target-immobilization strategy. RNA aptamer.	[59]
	MB	Aleutian mink disease virus (AMDV)	247 ± 62.5 nM	Enzyme-linked oligonucleotide assay (ELONA)	Prediction of the 3D structures using 3dRNA-v2.0.	Use of recombinant AMDV VP2 protein as a target. Monitoring the SELEX process by quantitative PCR (qPCR).	[60]
	MB	Rituximab	8.8 nM	ELISA using biotinylated aptamer	Prediction of 2D structure using Mfold. Structural analysis by circular dichroism (CD).	Target-immobilization strategy. Immobilization of rituximab on the protein A-coated MB for Fab orientation and easy detachment of target-bound aptamer from MB by heating at every round.	[61]
	MB	Human alpha-thrombin (Tb), human serum albumin (hSA)	1.2–20 nM (for hSA), 33 nM (for Tb)	MB-based qPCR method, ELONA, fluorescence evanescent wave biosensor.	Specificity using Cy3/biotinylated probe and MB-based fluorescence measurements. Prediction of 2D structure using Mfold.	Use of cross-linking reaction by binding between amino groups of target proteins and activated carboxylic acid groups of MBs. Short screening time (four cycles). Non-immobilization strategy.	[62]
	MB	Metronidazole	77.22 ± 11.27 nM	Fluorescence assay by labeling aptamer with FAM at the 5′-end.	Homology analysis by DNAMAN and Snap Gene. Prediction of secondary structures by RNA Structure software and Mfold Web Server. Simulation of tertiary structure by PyMoL 1.7.6 software.	Library-immobilized SELEX.	[63]
	Agarose bead	Flavin adenine dinucleotide (FAD)	0.61 ± 0.04 (for riboflavin), 0.44 ± 0.02 for FAD)	Monitoring the fluorescence of flavin after binding with aptamer.	Prediction of 2D structure using Mfold.	Library-immobilized SELEX.	[64]; Figure 1B
	Agarose bead	Butylone, ethylone, alpha-PVP	6.9 μM (for ethylone), 9.5 μM (butylone), 21 μM (PVP)	Isothermal titration calorimetry (ITC)	Specificity analysis by gel-elution assay.	Selection of multiple molecule–targeting aptamer.	[65]
	Agarose bead	Lucentis	25.72 ± 4.2 nM	Thermofluorimetric and non-faradaic impedance spectroscopy (NFIS) analysis.	Specificity analysis by NFIS analysis.	Use of stringent condition at each cycle for selecting aptamers with high affinity.	[66]
	Agarose bead	(−)-trans-Δ9-tetrahydrocannabinol (THC), UR-144 and XLR-11, two widely abused synthetic cannabinoids	61 ± 25 nM (for THC), 310 ± 70 nM (for XLR-11), 127 ± 32 nM (for UR-144)	ITC	Septicity analysis by strand-displacement fluorescence assay.	Library-immobilized strategy. A binding buffer containing organic solvent (i.e., 2.6% methanol or 5% DMSO) was used to increase their solubility.	[67]
	Agarose bead	3,4- methylenedioxypyrovalerone (MDPV)	6.1 ± 0.2 μM	ITC	Cy7-displacement assay	Library-immobilized strategy, immobilization of biotinylated cDNA:library duplex on bead.	[68]
	Agarose bead	N-Methyl Mesoporphyrin IX			Prediction of 2D structure using Mfold. Structural analysis by CD and PAGE.	Immobilization of biotinylated DNA:ssDNA duplex on streptavidin-coated agarose bead. Selection pressure: decrease of the concentrations of ssDNA and the target.	[69]
	Ni	N-cadherin derived from the human protein (Met1-Ala724)	93 nM	Fluorescence assay by labeling aptamer with FAM.		Immobilization of His-tagged protein on Ni-NTA column.	[70]; Figure 1C
Carbon nanomaterial–based platforms	GOx	*Vibrio parahaemolyticus*	10.3 ± 2.5 nM	Flow cytometry using 5′ FITC-labeled aptamer	Prediction of 2D structure using Mfold.	Non-immobilization strategy. Use of GOx functionalized with poly ethylene glycol and chitosan for enhanced water solubility and biocompatibility.	[71]; Figure 2A
	GOx	Okadaic acid (OA)	40 nM	Fluorescence assay by labeling aptamer with FAM at the 5′-end.	2D and 3D structure using RNA structure.	Screening of aptamer sequentially through three modes of SELEX. Use of oligo-free strategy during the early rounds, oligo-immobilizing strategy during last rounds, and counter-section strategy in the final rounds. Selection pressure: the decreasing amounts and shortening incubation time of ssDNA and OA reduced the probability of binding between ssDNA and OA.	[72]; Figure 2B
	GOx	Domoic acid (DA), saxitoxin (STX), and tetrodotoxin (TTX)	62.07 ± 19.97 nM (for DA), 44.12 ± 15.38 nM (for TTX), 61.44 ± 23.18 nM (for STX)	Use of FAM-labeled aptamer immobilized on GOx.	Prediction of 2D structure using Mfold.	Use of magnetic-reduced GOx (MRGO) platform for simple and specific partitioning. Employing the quenching property of GOx against fluorophore.	[51]; Figure 2C
	GOx	Thioflavin T	ND	ND	2D structure prediction by Nupack software. CD spectroscopy. Isothermal strand displacement amplification (SDA).	Non-immobilization strategy.	[73]
	GOx	Sulfaquinoxaline	82.54 nM	Fluorescence assay by labeling aptamer with TAMRA.	Prediction of 2D structure using Mfold.	Use of the quenching ability of GOx against fluorophore. Use of TMARA-labeled aptamer to monitor the optimization of SELEX.	[16]
Other nanomaterial-based platforms	AuNP	Zinc (II)-Protoporphyrin IX (ZnPPIX)	9.53 ± 1.86 μM	Fluorescence assay by labeling aptamer with FAM.	Prediction of 2D structure using Mfold. CD spectroscopy.	Optimization of adsorbing ssDNA library on AuNPs (60 mM NaCl and incubation time of 75 min).	[44]
	AuNP	Brassinolide (BL), bisphenol A (BPA)	17.3 nM (for BL), 37.9 nM (for BPA)	GNP-based colorimetric assays.	ITC. CD spectroscopy.	Monitoring of the progress of SELEX by salt-induced NP aggregation. Improvement of *K*_d_ value and specificity of truncated aptamer compared to full-length aptamer.	[74]; Figure 3A
	AuNP	Dichlorvos (DV)	42.3 nM	ITC, NanoZyme (mimic peroxidase activity of GNPs)-based colorimetric assay.	CD spectroscopy. Determination of primary sequence homology among candidate aptamers using CLUSTALW. 2D structure prediction by Nupack software.	Aptamer-NanoZyme (AuNP having peroxidase mimic activity)-based colorimetric assay, colorimetry (AuNP, TMB), nanozyme.	[75]
	Magnetic nanoparticle (MNP)	Lipopolysaccharides (LPS)	102 ± 17 nM	Fluorescence assay by labeling aptamer with FAM.	2D structure prediction by DNAMAN 8.	Library-immobilization strategy.	[76]
	MNP	RNase B, transferrin	91 ± 30 nM (for RNase B), 88 ± 31 nM (for transferrin)	Capillary electrophoresis (CE) equipped with laser-induced fluorescence (LIF) assay.	Selectivity analysis by CE-LIF assay.	Use of glycan-imprinted MNP. Target-immobilization strategy.	[77]
	MNP	Myoglobin (Mb), β2-microglobulin	46.3 ± 10.4 nM (for Mb), 36.7 ± 11.4 nM (for β2-microglobulin)	Enzyme-linked oligosorbent assay	Enzyme-linked oligosorbent assay.	Use of epitope-imprinted MNP. Target-immobilization strategy. Short screening time (three cycles, takes 1 day).	[78]; Figure 3B
	MNP	Saponin (ginsenoside Re and Rb1)	2.3 ± 0.3 μM (for Re), 4.9 ± 0.3 μM (for Rb1)	Fluorescence assay by labeling aptamer with FAM	2D structure analysis.	Use of polyethyleneimine-assisted boronate affinity MNPs.	[79]
	Silver nanoparticle (AgNP)	Lactoferrin (Lac)	0.953 ± 0.114 nM	Surface plasmon resonance measurement.	Specificity using surface plasmon resonance imaging, structure prediction by Oligoanalyzer 3.1.	Use of AgNP-conjugated oligos. Target immobilization on microarray. Enabled real-time monitoring of binding properties and specificity between the target and the aptamer.	[80]; Figure 3C

ND, not determined.

**Table 3:** Advantages and limitations of micro/nanomaterial-based platforms.

Classification	Material	Advantage	Limitation
Bead-based platform	Magnetic bead	Simple partitioning of unbound and bound oligos using magnet	Required a conjugation process for target or oligo-immobilization strategy
	Agarose bead	Enables a microcolumn bead-based SELEX Reducing non-specific binding	Limitation of high-throughput selection
	Nickel bead	Enables a microcolumn bead-based SELEX Reducing non-specific binding enables a relatively free interaction of target and oligos by His-tag for lifting targets on solid substrate	Need for additional His-tagging of target
Carbon-based nanomaterial platform	Graphene oxide (GOx)	Enables a free interaction of target and oligo Simple removal of unbound oligos using selective adsorption of ssDNA by GOx Enables fabrication of GOx with additional functionality (e.g., magnetic reduced GOx) Enables optimization of SELEX conditions using the fluorescent quenching property of GOx	Potential failure of separation by low water solubility
	Gold nanoparticle	Enables a free interaction of target and oligo Simple removal of unbound oligos using selective adsorption of ssDNA by GOx Enables real-time monitoring SELEX process via color change by salt-induced AuNP aggregation	Required a conjugation process for target
	Magnetic nanoparticle	Simple partitioning of unbound and bound oligos using magnet	Need for a conjugation process for target or oligo-immobilization strategy
	Silver nanoparticle	Enables a development of SELEX strategy combined with other plasmonic sensing strategy (e.g., SPRi)	Required a conjugation process for target

**Table 4:** Software used for the characterization of aptamers.

Purpose	Software	Features	Website	Reference
Aptamer-protein interaction prediction	AptaNet	Use of balancing technique and a deep neural network	https://github.com/nedaemami/AptaNet (accessed on 24 August 2021)	[101]
	PPAI	Use of the abstracted sequence features and the machine learning framework	http://39.96.85.9/ PPAI (accessed on 24 August 2021)	[102]
RNA-protein interaction	RPINBASE	Predicting the interactions between RNAs and proteins by applying machine learning approaches	http://rpinbase.com/Explore (accessed on 24 August 2021)	[103]
Aptamer 3D structure prediction	RNAComposer	Based on the machine translation principle and operates on the RNA FRABASE database	http://rnacomposer.cs.put.poznan.pl/(accessed on 24 August 2021)	[104]
	3dRNA	Automated method of building RNA 3D structures from sequences and 2D structures by using the smallest secondary elements.	http://biophy.hust.edu.cn/3dRNA (accessed on 24 August 2021)	[105]
	Vfold3D	Template-based coarse-grained structure prediction model	http://rna.physics.missouri.edu/ vfold3D/ (accessed on 24 August 2021)	[106]
	simRNA	Simulations of RNA conformational dynamics (folding, unfolding, multiple chain complex formation, etc.)	https://genesilico.pl/SimRNAweb (accessed on 24 August 2021)	[107]
	ToGo-WF	Prediction of RNA 3D structures and RNA-RNA/protein interactions using the KNIME workflow	https://togo.medals.jp/active_local_rna_prediction.eng.html (accessed on 24 August 2021)	[108]
Aptamer 2D structure prediction	ViennaRNA	Calculation of either minimum free energy or partition functions	https://www.tbi.univie.ac.at/RNA/ (accessed on 24 August 2021)	[109]
	Mfold	Use of free energy minimization method	http://www.unafold.org/ (accessed on 24 August 2021)	[110]
	RNAsoft (AveRNA)	Energy-based, pseudoknot-free RNA secondary structure prediction	http://www.rnasoft.ca/ (accessed on 24 August 2021)	[111]
	RNAstructure	Use of free energy minimization method	https://rna.urmc.rochester.edu/RNAstructureWeb/ (accessed on 24 August 2021)	[112]
	NUPACK	Calculation of the partition function and minimum free energy secondary structure	http://www.nupack.org/ (accessed on 24 August 2021)	[113]
Molecular docking	ZDOCK	Fast Fourier transform (FFT) algorithm to search and obtain all the binding poses	https://zdock.umassmed.edu/ (accessed on 24 August 2021)	[114]
	MDockPP	FFT algorithm to collect all putative binding poses	https://zougrouptoolkit.missouri.edu/MDockPP/ (accessed on 24 August 2021)	[115]
	AutoDock	Calculation of the free energy to score binding poses	http://autodock.scripps.edu/ (accessed on 24 August 2021)	[116]
	AutoDock Vina	Use of an empirical scoring function to score the binding poses	http://vina.scripps.edu/ (accessed on 24 August 2021)	[117]

## Data Availability

No new data were created in this study. Data sharing is not applicable to this article.
